# International Union of Basic and Clinical Pharmacology. CXXII. Applying an objective evaluation to the status of class A orphan G protein-coupled receptors

**DOI:** 10.1016/j.pharmr.2026.100141

**Published:** 2026-05-08

**Authors:** Stephen P.H. Alexander, Kirstie A. Bennett, Andrew J. Brown, Michelle Glass, David E. Gloriam, Julien Hanson, Paul A. Insel, Eamonn Kelly, Christopher J. Langmead, Kirill A. Martemyanov, Rick R. Neubig, Stefan Offermanns, Mette M. Rosenkilde, Gunnar Schulte, Nicola J. Smith, Huixian Wu, Elena Faccenda, Simon D. Harding, Anthony P. Davenport

**Affiliations:** 1 Division of Physiology, Pharmacology and Neuroscience, Life Sciences, University of Nottingham Medical School, Nottingham, United Kingdom; 2 Nxera Pharma UK Ltd, Cambridge, United Kingdom; 3 Ikherma Consulting Ltd, Hertfordshire, United Kingdom; 4 School of Pharmacy and Pharmacology, University of Otago, Otago, New Zealand; 5 Department of Drug Design and Pharmacology, University of Copenhagen, Copenhagen, Denmark; 6 Laboratory of Molecular Pharmacology, GIGA Research Institute and Center of Interdisciplinary Research on Medicine, University of Liège, Liège, Belgium; 7 Departments of Pharmacology and Medicine, University of California, San Diego, La Jolla, California; 8 School of Physiology, Pharmacology and Neuroscience, University of Bristol, Bristol, United Kingdom; 9 Drug Discovery Biology and Neuromedicines Discovery Centre, Monash Institute of Pharmaceutical Sciences, Monash University, Parkville, Victoria, Australia; 10 Department of Physiology and Biophysics, University of Miami Miller School of Medicine, Miami, Florida; 11 Department of Pharmacology and Toxicology, Michigan State University, East Lansing, Michigan and Nicholas V. Perricone, MD, Division of Dermatology, Department of Medicine, College of Human Medicine, East Lansing, Michigan; 12 Department of Pharmacology, Max Planck Institute for Heart and Lung Research, Bad Nauheim Germany and Center for Molecular Medicine, Goethe-University Frankfurt, Frankfurt am Main, Germany; 13 Department of Biomedical Sciences, Lab for Molecular Pharmacology, University of Copenhagen, Copenhagen, Denmark; 14 Department Physiology and Pharmacology, Section of Receptor Biology and Signaling, Karolinska Institutet, Stockholm Sweden; 15 Orphan Receptor Laboratory, Department of Pharmacology, School of Biomedical Sciences, Faculty of Medicine & Health, UNSW Sydney, Sydney, Australia; 16 Medicine Design, Pfizer Research and Development, Pfizer Inc, Groton, Connecticut; 17 Institute for Neuroscience and Cardiovascular Research, College of Medicine & Veterinary Medicine, University of Edinburgh, Edinburgh, United Kingdom; 18 Experimental Medicine and Immunotherapeutics, Department of Medicine, University of Cambridge, Level 6, Centre for Clinical Investigation, Addenbrooke's Hospital, Cambridge, United Kingdom

## Abstract

In this review, we, on behalf of the Nomenclature and Standards Committee of the International Union of Basic and Clinical Pharmacology, describe criteria for assessing the evidence for pairing receptors with endogenous/physiological ligands for formal receptor deorphanization. This process is illustrated through consideration of the class A G protein-coupled receptors (GPCRs) not yet formally paired with an endogenous/physiological ligand by the Nomenclature and Standards Committee of the International Union of Basic and Clinical Pharmacology. Of the 67 orphan class A GPCRs considered, 25 class A GPCRs have no identified endogenous agonists, although 5 (*GPR21, GPR27, GPR52, GPR85,* and *GPR88*) have synthetic ligands that have the potential to be used as tools for uncovering physiological roles and further pharmacological properties of these receptors. Surprisingly, 6 orphan GPCRs (*GPR135*, *GPR152*, *GPR153*, *MRGPRF*, *MRGPRG*, and *MRGPRX3*) have no clear pharmacology or phenotype reported following genetic disruption. Thirty-two orphan GPCRs have been paired with at least 1 endogenous agonist (mainly lipids and their derivatives, peptides, and other metabolites), but further characterization is required from the scientific community to validate these results. We identify 10 orphan class A GPCRs for which there are plausible grounds for considering deorphanization: *GPR4* (protons), *GPR15* (GPR15L), *GPR31* (12S-hydroxyeicosatetraenoic acid), *GPR39* (zinc divalent ions, Zn^2+^), *GPR65* (protons), *GPR68* (protons), *GPR132* (9-hydroxyoctadecadienoic acid), *GPR183* (7*α*,25-dihydroxycholesterol), *MRGPRD* (*β*-alanine), and *MRGPRX1* (bovine adrenal medulla peptide 8-22). The issue of nomenclature for these 10 GPCRs will be considered by further subcommittees of the Nomenclature and Standards Committee of the International Union of Basic and Clinical Pharmacology. We hope this review will prompt further investigations into these members of the currently most widely clinically exploited protein superfamily.

**Significance Statement:**

The use of systematic, rational nomenclature for drug targets provides a framework to ensure consistent identification and rapid recognition. Given that G protein-coupled receptors have fundamental physiological roles and are widespread targets of drugs in current clinical use, we hope the target summary and deorphanization criteria provided here will prompt renewed efforts to investigate these orphan receptors as regulators of physiology and as opportunities for future therapeutic exploitation.

## I Introduction and scope

NC-IUPHAR is the Nomenclature and Standards Committee of the International Union of Basic and Clinical Pharmacology (https://www.guidetopharmacology.org/nciuphar.jsp). One of the central objectives of NC-IUPHAR is to provide guidelines for the nomenclature and classification of biological targets of drugs, which we present through an online, freely accessible data base at www.guidetopharmacology.org. The intention of the NC-IUPHAR-recommended nomenclature is to provide guidance on the naming of drug targets that is clear, logical, and consistent so that the broader scientific community avoids confusion and saves time and energy.

NC-IUPHAR has previously published a G protein-coupled receptor (GPCR) list,^1^ which was updated to reflect preliminary pairings between orphan GPCRs and endogenous ligands, providing initial criteria for deorphanization.^2^ In this reflection on the current state of this field of research, we update the criteria that NC-IUPHAR applies to progress the formal deorphanization of a receptor and use the class A GPCRs to illustrate the process.

Over the last 30 years, many publications from NC-IUPHAR have appeared in *Pharmacological Reviews* and have identified the preferred nomenclature for a number of families of class A GPCRs (Table 1). In the absence of a formal publication, NC-IUPHAR has established a nomenclature for several families communicated via the website www.guidetopharmacology.org. These include the bile acid receptor, dopamine receptors, galanin receptors, glycoprotein hormone receptors, gonadotropin-releasing hormone receptors, melanin-concentrating hormone receptors, melanocortin receptors, motilin receptor, neuromedin U receptors, neuropeptide FF/neuropeptide AF receptors, neuropeptide S receptor, neuropeptide W/neuropeptide B receptors, neurotensin receptors, opioid receptors, platelet-activating factor receptor, prokineticin receptors, prolactin-releasing peptide receptor, QRFP receptor, tachykinin receptors, thyrotropin-releasing hormone receptors, and vasopressin and oxytocin receptors (Table 1).

**Table 1:** Human class A G protein-coupled receptor families, which the Nomenclature and Standards Committee of the International Union of Basic and Clinical Pharmacology considers have an established endogenous/physiological ligand

**Family**	**Family**
5-Hydroxytryptamine receptors^88^^,^^89^	Melanin-concentrating hormone receptors
Acetylcholine receptors (muscarinic)^90^	Melanocortin receptors
Adenosine receptors^91^^,^^92^	Melatonin receptors^93^
Adrenoceptors^94^^,^^95^	Motilin receptor
Angiotensin receptors^96^^,^^97^	Neuromedin U receptors
Apelin receptor^98^^,^^99^	Neuropeptide FF/neuropeptide AF receptors
Bile acid receptor	Neuropeptide S receptor
Bombesin receptors^100^	Neuropeptide W/neuropeptide B receptors
Bradykinin receptors^101^	Neuropeptide Y receptors^102^
Cannabinoid receptors^83^^,^^103^	Neurotensin receptors
Chemerin receptors^104^	Opioid receptors
Chemokine receptors^3^^,^^105^, ^106^, ^107^	Orexin receptors^108^^,^^109^
Cholecystokinin receptors^110^	Oxoglutarate receptor^2^
Complement peptide receptors^111^	P2Y receptors^112^
Dopamine receptors	Platelet-activating factor receptor
Endothelin receptors^113^^,^^114^	Prokineticin receptors
Formylpeptide receptors^85^	Prolactin-releasing peptide receptor
Free fatty acid receptors^115^	Prostanoid receptors^116^, ^117^, ^118^
G protein-coupled estrogen receptor^119^	Proteinase-activated receptors^82^
Galanin receptors	QRFP receptor
Ghrelin receptor^120^	Relaxin family peptide receptors^121^^,^^122^
Glycoprotein hormone receptors	Somatostatin receptors^123^
Gonadotropin-releasing hormone receptors	Succinate receptor^2^
Histamine receptors^124^^,^^125^	Tachykinin receptors
Hydroxycarboxylic acid receptors^126^	Thyrotropin-releasing hormone receptors
Kisspeptin receptor^127^	Trace amine receptor^128^
Leukotriene, lipoxin and 5-oxoeicosatrienoic acid receptors^129^, ^130^, ^131^	Urotensin receptor^132^
Lysophospholipid (LPA) receptors^8^^,^^9^	Vasopressin and oxytocin receptors
Lysophospholipid (S1P) receptors^8^^,^^9^	

The citations indicated are IUPHAR nomenclature publications in Pharmacological Reviews.

A further 17 class A orphan GPCRs are associated with NC-IUPHAR subcommittees and may be regarded as ”foster children” (Table 2). During the course of these discussions, the chemokine receptor subcommittee of NC-IUPHAR has suggested renaming *GPR182* as the newest member of the atypical chemokine receptor subfamily, ACKR5.^3^ We highlight this group of GPCR “foster children” to identify that, in the near future, NC-IUPHAR subcommittee panels will apply the systematic criteria we identify here to assess the status of these GPCRs in more detail.

**Table 2:** Seventeen human class A orphan GPCR associated with the indicated NC-IUPHAR subcommittees (“foster children”)

NC-IUPHAR subcommittee/s	Target/s
Angiotensin receptors	*Mas1*
Bombesin receptors	*BB*_*3*_*receptor*
P2Y receptors Leukotriene receptors	*GPR17*
Cannabinoid receptors	*GPR18* *GPR55* *GPR119*
Chemokine receptors	*GPR182*
Free fatty acid receptors	*GPR42* *GPR84*
Leucine-rich repeat–containing receptors	*LGR4* *LGR5* *LGR6*
Trace amine receptors	*TAAR2* *TAAR5* *TAAR6* *TAAR8* *TAAR9*

NC-IUPHAR, Nomenclature and Standards Committee of the International Union of Basic and Clinical Pharmacology.

Here, we evaluate the remaining group of 67 Class A GPCRs for their orphan status in a systematic, pragmatic manner.

## II Class A G protein-coupled receptor lacking a proposed endogenous/physiological ligand

Although there are data regarding the molecular nature and tissue expression levels of 26 class A GPCRs (Table 3), currently, there are no published data suggesting endogenous/physiological ligands that may activate them. Synthetic ligands have been reported for 5 orphan GPCRs (*GPR21, GPR27, GPR52, GPR85*, and *GPR88*), although these still require independent validation (Table 3). By contrast, 6 of these orphan GPCRs (*GPR135*, *GPR152*, *GPR153*, *MRGPRF*, *MRGPRG*, and *MRGPRX3*) have no reported pharmacology and no phenotype for genetic disruption in the public domain. *GPR135*, *GPR152*, *MRGPRE*, and *MRGPRG* were expressed and screened against over 5000 endogenous compounds without identifying any hits.^4^ The Human Protein Atlas has combined bulk RNA sequencing data from 3 databases, but mRNA encoding *GPR152* and *MRGPRG* was not detected in 50 human tissues or organs. However, low levels of *GPR152* mRNA were reported following single-cell sequencing in endothelial cells and mononuclear phagocytes for the human heart. *MRGPRG* expression was also reported in specialized epithelial and germ cells using this technique. Only mRNA encoding *GPR135* and *GPR153* was reported to be widely distributed, although at low levels, with some evidence of protein expression using immunocytochemistry. *GPR135* mRNA was reported to be present in human cancers using polymerase chain reaction.^5^ *MRGPRE* is reported in the brain and female reproductive organs, whereas *MRGPRX3* is reported in the salivary glands. Other members of the Mas-related GPCR family were originally identified in sensory neurons, but more information is needed regarding the precise cellular expression of mRNA and protein that may indicate potential roles in humans.

**Table 3:** Twenty-five human class A orphan GPCR for which there are currently no identified endogenous/physiological ligands

Target	Reported Signaling	Surrogate Ligands	Phenotype of Gene Disruption^a^	Comments
*GPR20*	Reported to couple constitutively to G_i_^80^^,^^133^	-	-	A cryo-EM structure of the recombinant *GPR20* was suggested to compare with galanin receptors; the authors interpreted their observations to suggest the N terminus was able to activate the receptor in the absence of other ligands^134^
*GPR21*	Reported to couple constitutively to G_q_^135^	GRA2 is reported to act as an inverse agonist^136^	Positive benefit in models of diabetes/obesity^137^^,^^138^ were not observed in another study^139^	A complex mix of plasmalogens evoked phosphorylation of ERK and protein kinase B in mouse cultured hippocampal neurones that could be inhibited with shRNA directed against *GPR21*^140^ A cryo-EM structure of unliganded *GPR21* bound to G proteins has been reported^141^
*GPR22*	Reported to couple constitutively to G_i_^142^^,^^143^	-	An increased susceptibility to cardiac decompensation following aortic banding^142^	-
*GPR26*	Reported to couple constitutively to G_s_^144^	-	Increased symptoms of anxiety and depression^145^, hyperphagia and adiposity^146^	-
*GPR27*	Reported to couple constitutively to G_i_^80^	5128535^147^, ^148^, ^149^ and PT-91^149^ functioned as surrogate agonists, whereas Compound 1a was reported as an inverse agonist,^150^ although a further study was unable to confirm this activity^148^	A modestly reduced insulin gene transcription without altering glucose tolerance^151^ Zebrafish showed altered insulin signaling and metabolite levels^152^	A complex mix of plasmalogens evoked phosphorylation of ERK and protein kinase B in mouse cultured hippocampal neurones that could be inhibited with shRNA directed against *GPR27*^140^
*GPR33*	Heterologous expression of the mouse receptor resulted in elevated inositol phosphates levels^153^	-	-	*GPR33* has been reported to bind radiolabeled cortisol.^154^ Human *GPR33* contains a premature stop codon within the coding sequence of the second intracellular loop^153^ Pseudogenes of *GPR33* predominantly occur in rodents (rat) and primates but not other mammals.^153^ Receptor inactivation may have occurred due to a rodent–hominoid-specific pathogen^155^ Proposed to act as a viral anchoring site^156^
*GPR45*	Reported to couple constitutively to G_i_^80^ and to G_s_^157^	-	Increased adiposity at weaning and reduced hypothalamic expression of *POMC*.^158^ Global disruption of *Gpr45*^159^ or selectively in the paraventricular nucleus of the hypothalamus was associated with obesity^157^	-
*GPR52*	Reported to recruit *β*-arrestin constitutively in an inactive state^160^	A number of synthetic agonists have been described, including compound 7a and 7m,^161^ FTBMT,^162^ derivative 17,^163^ BD442618, BD502657,^164^ HTL0041178,^165^ and sucralose^166^ Additionally, multiple antagonists/inverse agonists have been reported, including cannabidiol, O1918,^167^ and comp-43^168^	Elevated locomotor response to the A_2A_ adenosine receptor antagonist istradefylline^169^	A crystal structure of GPR52 with derivative 17 bound has been reported, with an interpretation that the ligand might act allosterically^170^
*GPR62*	Reported to couple constitutively to G_s_^171^	-	No overt phenotype^171^ or impact on CNS myelination^172^	Reported to inhibit MT_2_ receptor coupling to *β*-arrestin^173^
*GPR78*	Reported to couple constitutively to cAMP production^144^	-	Overexpression of *GPR78* in mice was associated with a salivary gland dysfunction^174^	Pubmed gets *GPR78* confused with a 78 kDa glucose-regulated protein known as the endoplasmic reticulum chaperone BiP
*GPR82*	Reported to lack constitutive activity in recombinant expression^80^	-	A reduced body fat and weight, with increased insulin sensitivity and glucose tolerance^175^	Although lacking constitutive activity in recombinant expression,^80^ LysoPE and LysoPC were reported to act as inverse agonists^176^
*GPR85*	Reported to lack constitutive activity in recombinant expression^80^	Compound 1a^150^ and compound 3i^177^ are reported as inverse agonists Harmine has been reported to act as an inverse agonist^178^	Mild overexpression was associated with reduced brain mass, whereas increased brain mass was found in knockout animals^179^	-
*GPR88*	Reported to couple to G_i_^143^^,^^180^^,^^181^	A number of small molecule agonists have been reported including 2-PCCA,^b^^,^^182^ RTI-122,^183^ RTI-13951-33,^184^^,^^185^ compound 2 [PMID: 24793972],^181^ compound 99,^186^ and BI-9508^187^	Gene disruption resulted in altered striatal signaling,^188^ reduced adiposity and reduced spontaneous food intake,^189^ increased alcohol intake^190^	A cryo-EM structure of GPR88 combined with G_i1_ has been described, where 2-PCCA bound as an allosteric modulator^191^ Reported to inhibit signaling through *μ* and *δ* opioid receptors^192^^,^^193^
*GPR135*	Reported to recruit *β*-arrestin constitutively, but failed to influence G_i_/G_s_ pathways^173^	-	-	Reported to inhibit MT_2_ receptor coupling to *β*-arrestin^173^
*GPR141*	-	-	Elevated immune response in leukocytes and experimental autoimmune encephalomyelitis^194^	-
*GPR148*	-	-	-	No orthologue exists in rodents
*GPR149*	-	-	Increased fertility in male mice,^195^ reduced oligodendrocyte progenitor cell maturation,^196^ and reduced weight gain on high-fat diet^197^	-
*GPR150*	Reported to couple constitutively to G_i_^80^	-	-	-
*GPR152*	-	-	-	-
*GPR153*	Inferred to couple to G_i_ in a manner insensitive to pertussis toxin^198^	-	Reduced inflammatory signaling in models of stroke and experimental autoimmune encephalitis^198^	-
*GPR161*	Appears to function as a negative regulator of Sonic hedgehog signaling^199^, ^200^, ^201^, ^202^	-	Reduced embryonic ciliary function^203^	Variants of *GPR161* are associated with developmental disorders in man^204^^,^^205^ A cryo-EM structure suggests the second extracellular loop may auto-activate the receptor.^81^ Reported to act as a mechanosensor in neuronal cilia^206^
*GPR176*	Reported to couple constitutively to G_i_,^80^ to G_z_ in the suprachiasmatic nucleus^207^ and to G_s_ in colorectal cancer cells^208^	-	Knockout mice appeared superficially normal, but exhibited a shorter circadian period^207^	Shows circadian variation in the suprachiasmatic nucleus.^207^ Activation of *GPR176* was observed after co-incubation with a microwaved supernatant fraction from mouse brain^209^
*MRGPRF*	-	-	-	Ang-1-7 was reported to inhibit arachidonic acid release in the presence of *MRGPRF*,^210^ although there was a lack of clarity whether this was an agonist or inverse agonist function
*MRGPRG*	-	-	-	-
*MRGPRX3*	-	-	-	*Mrgprx3* is a pseudogene in mice

CNS, central nervous system.

a Unless noted otherwise, the phenotype is derived from mouse models.

b (1R, 2R)-2-pyridin-2-yl-cyclopropane carboxylic acid ((2S, 3S)-2-amino-3-methyl-pentyl)-(4'-propylbiphenyl-4-yl)-amide.

One of the aspirations of this review is to highlight opportunities for further research. Given the wealth of drugs in clinical use that target GPCRs, in particular, from class A,^6^ we suggest that additional investigation of these 26 receptors could potentially lead to useful therapeutic opportunities.

## III Developing criteria for the deorphanization process

The remaining 41 GPCRs were considered to have sufficient published data to be subject to closer scrutiny. The expert reviewers (taken from the listed co-authors) were recruited based on their experience with the topic, while also reflecting diversity in geography and gender, as well as representing both academic and industrial roles. Each reviewer was allocated three GPCRs as a primary reviewer and 6 GPCRs as a second reviewer, with an aim to get an independent (unbiased) evaluation and triangulation of the status of those GPCRs. Reviewers were not allocated orphan GPCRs where they had published original articles on that receptor.

The aim for the primary reviewers was to research the 3 assigned GPCRs and to update and/or correct the records in www.guidetopharmacology.org. In addition, primary reviewers summarized the evidence according to the evaluation checklist shown in Table 4. Secondary reviewers were not expected to research their topics to the same depth as primary reviewers but to accumulate sufficient evidence to make a judgment as to a 3-option outcome (strong, intermediate, and weak deorphanization cases evaluated as *α*, *β*, or *γ*—Table 5). For each receptor, at least 3 reviews were received. GPCRs were then ranked, both in terms of the judged strength of evidence and the convergence of evaluation from the reviewers. Thereafter, online sessions were conducted with the reviewers divided into 2 geographical sections, which allowed free discussions on the grading of the candidate GPCR across the entire panel of experts.

**Table 4:** Evaluation checklist used by reviewers of orphan G protein-coupled receptor

1	The crux	What is/are the suggested putative endogenous agonist/s?
2	Reproducibility	a Has the activity of this putative agonist been reproduced in the same signaling paradigm by a fully independent laboratory?^a^
		b Has the receptor been included in a published major screening program and with what outcome?^b^
3	Physiology	c Is the potency of the putative agonist consistent with a physiological function?
		d Are there plausible mechanisms for the putative agonist to reach bioactive concentrations in tissues expressing the receptor? Is the ligand:receptor pairing physiologically feasible?
4	Binding	Has binding of the putative agonist to the receptor been demonstrated?^c^
5	Agonist effects	e What responses does the putative agonist evoke in cells expressing the recombinant receptor?^d^
		f What responses does the putative agonist evoke in native cells/tissues expressing the receptor?
		g Does the putative agonist evoke a response in the absence of the receptor (off-target effects)?
6	Pharmacology	h If synthetic agonists have been described, how closely do they mimic the effects of the putative agonist?^e^
		i Are there antagonists that block the effects of the putative agonist (and/or synthetic agonists)?
		j Does inhibition (pharmacological or genetic) of the enzymes capable of producing or terminating the putative agonist alter the receptor’s downstream signaling?
7	Genetic approaches	k Do genetic alterations (natural mutations/single nucleotide polymorphisms or man-made), which alter receptor sequence or expression levels, disrupt cellular/tissue/behavioral responses to the putative agonist?
		l In the case of peptide ligands, do genetic alterations of the coding gene result in changes in receptor expression?

LysoPE, lysophosphatidylethanolamine.

a Were cells/cDNA independently derived?.

b For example, Southern et al.^4^

c This might be displacement of labeled ligands or biophysical evidence. Although this is desirable, it would not be considered obligatory as it is often very challenging.

d Calcium elevations, *β*-arrestin recruitment, cAMP inhibition, receptor internalisation, etc.

e Consider the possibility of biased agonism.

**Table 5:** Evaluation options identified by reviewers of orphan G protein-coupled receptor

*α*.	Enough evidence from independent laboratories to establish a strong case for deorphanization
*β*.	Remains an orphan receptor with some evidence for endogenous ligands and/or function, but requires independent confirmation
*γ*.	Remains an orphan receptor with limited evidence for function

Of the criteria against which the receptors were compared, some are fundamental elements, such as the identification of a putative endogenous agonist (point 1) and the independent confirmation process (point 2). Point 2b identifies that literature searching for individual GPCRs may overlook mass screening investigations, such as Southern et al.^4^ Others relate to the physiological confirmation of the endogenous agonist (point 5) and whether tool compounds exist to dissect the pharmacological profile of the receptor (point 6). Genetic modification of the receptor, either as naturally occurring variants/single nucleotide polymorphisms or by molecular disruption (point 7), also provides evidence for the functional impact of the receptor.

Of these criteria, it is worthwhile to reflect on 2 specific points. First, identifying binding of the putative endogenous ligand to the receptor (point 4) can be challenging for a number of reasons. The generation of a radiolabeled analog may be tricky in terms of synthetic chemistry. Fluorescent analogs may be equally challenging to synthesize. Additionally, many endogenous ligands bind at high nanomolar to low micromolar concentrations, which are difficult to quantify using conventional radioligand binding techniques. The likelihood of the ligand binding to other cellular components could also be a factor in limiting the levels of specific binding. We consider demonstration of binding of the putative endogenous agonist not to be an essential prerequisite, although it is a desirable confirmation.

A more impactful criterion is whether there is the capacity to manipulate tissue levels of the putative endogenous ligand through modifying the enzyme activities involved in the synthesis or degradation/transformation of the ligand (point 6c). A requirement for this step is obviously that these enzyme activities are known and that inhibitors are available. Although this criterion is also a high bar to overcome, it allows distinction between ligands that can activate the receptor in vitro and those that really activate the receptor *in situ*.

## IV Orphan class A G protein-coupled receptors with a proposed endogenous/physiological ligand

On the basis of triangulated reviews from the expert panel using these criteria, 31 GPCRs were identified as having limited evidence for deorphanization (Table 6). The most common reasons for this conclusion included a lack of confirmation by independent laboratories and questions about the physiological levels of the putative endogenous ligand/s in close proximity to the receptor, but particularly the “high bar” of the impact of manipulating levels of the putative endogenous agonist on receptor responsivity (point 6c).

**Table 6:** Thirty-two human class A orphan G protein-coupled receptor with reported putative endogenous/physiological agonists identified as having weak potential (rated *β* or *γ* by the reviewers) for deorphanization at the current time

Target	Proposed Physiological Agonist/s	Surrogate Agonist/s	Antagonists/Inverse Agonists	Comment
*GPR3*	S1P^211^ Oleic acid,^212^ ODA, and OEA^212^^,^^213^	Diphenyleneiodonium^212^^,^^214^^,^^215^ and analogs^216^	AF64394^212^^,^^217^ and cannabidiol^218^ are reported to act as inverse agonists	Activation by S1P was not repeated in *β*-arrestin assays^4^^,^^20^ Fluorescent analogs of AF64394 have been reported to bind to the recombinant receptor^219^ AF64394 is reported to bind to the membrane interface of a GPR3 homodimer based on cryo-EM studies^220^
*GPR6*	S1P^211^^,^^221^ Palmitoleic acid^212^	-	Cannabidiol,^218^ CVN264^222^, ^223^, ^224^, ^225^, CVN-424 (Solengeprase)^226^ and CVN527^227^ are reported to act as inverse agonists	Activation by S1P was not repeated in *β*-arrestin assays^4^^,^^20^ Reported to form functional heterodimers with LPA and S1P receptors^228^
*GPR12*	S1P^211^^,^^221^ SPC^229^ Oleic acid^212^	Tyrosol^230^ Multiple fungal-derived steroid-like compounds^231^	Cannabidiol^232^ is reported to act as an inverse agonist	Activation by S1P was not repeated in *β*-arrestin assays^4^^,^^20^
*GPR19*	Adropin^233^^,^^234^	-	-	Adropin was not effective in *β*-arrestin assays^4^^,^^20^ Knockdown of *Gpr19* in H9c2 rat cardiac cells^235^ or RAW264.7 mouse monocyte/macrophage cells^236^ resulted in a reduced responsiveness to adropin. Similarly, siRNA targeting of *GPR19* in human primary fibroblasts attenuated the effects of adropin^237^
*GPR25*	CXCL17^238^^,^^239^	-	-	Reported to couple constitutively to G_i_.^80^ Nonmammalian orthologues are reported to be activated by apelin and apela^240^
*GPR32*	RvD1^241^^,^^242^ Lipoxin A_4_^242^ Resolvin D3^243^	Compound 43^242^ Multiple chemotypes were identified in a high-throughput screening assay^241^	An antibody directed against *GPR32* blocked RvD1-evoked responses in isolated human polymorphonuclear leukocytes^244^ and blocked RvD3-evoked responses in a recombinant *GPR32*-expressing cell line^243^	Studies in macrophages that express *GPR32* but not FPR2/ALX suggest RvD1 induced a pro-resolution phenotype (increased phagocytosis, blocked chemotaxis, reduced secretion of proinflammatory cytokines IL-1*β*, IL-8, and MCP-1). This effect was reduced with siRNA against *GPR32* and was consistent with RvD1 as a pro-resolution molecule.^245^ RvD1 was not effective in *β*-arrestin assays,^4^^,^^20^ or in dynamic mass redistribution or internalization assays.^39^ *Gpr32* is a pseudogene in mice and rats^246^ Radioligand binding studies have been performed in a cell line that expresses the FPR1/ALX receptor, which is also a receptor for RvD1, and binding of RvD1 to *GPR32* was inferred due to a binding remaining in the presence of FPR2/ALX agonist^242^
*GPR34*	LysoPS^247^, ^248^, ^249^, ^250^	Compound 14 was reported as a high-potency synthetic agonist with selectivity relative to *P2RY10*^251^ Compound M1 exhibited high potency in an assay for TGF-*α* shedding^250^ M1 effects in primary microglia were lost following *Gpr34* knockdown^252^	YL-365^253^ An antagonist has been claimed to block the effects of LysoPS in cultured cells, but a structure or source was not reported^254^ (*S*)-3-(4-(benzyloxy)phenyl)-2-(2-phenoxyacetamido) propanoic acid derivatives^255^ Aminoethyl-carbamoyl ATP was identified as an antagonist at the carp orthologue^248^ Compound D2^256^	The stimulatory effects of LysoPS were not reproduced in alternative studies^4^^,^^20^^,^^257^ Genetic disruption of iPLA2-G6 reduced LysoPS and mimicked the effects of *Gpr34* knockdown in male mouse hearts^258^ Cryo-EM structures of LysoPS bound to *GPR34* have been described^250^^,^^253^^,^^259^
*GPR35*	Kynurenic acid^4^^,^^260^, ^261^, ^262^ 2-oleoyl-LysoPA^263^ 5,6-dihydroxyindole-2-carboxylic acid^264^ CXCL17^265^ 5HIAA^266^	Z^261^^,^^262^^,^^267^, ^268^, ^269^ NPPB^261^^,^^270^ pamoic acid^261^^,^^262^^,^^269^ cromoglicic acid^264^^,^^268^^,^^269^ nedocromil^264^^,^^268^ entacapone^262^ tyrphostin-51^262^ furosemide^271^ bumetanide^271^ benserazide^272^ pyrogallol^272^ compound 83^273^ lodoxamide^274^ Isobavachin^275^ Pelargonidin^276^	CID2745687^261^^,^^273^ ML145^272^^,^^277^	There are large interspecies pharmacology differences between rat, mouse, and human *GPR35*. Kynurenic acid and zaprinast are up to 100-fold less potent at human than rat *Gpr35*, whereas pamoate is more active at the human receptor^269^ The antagonists described are also higher affinity at the human orthologue compared to rodent receptors Humans have 2 isoforms of *GPR35,* whereas rodents have only one^278^ Isoforms show slightly different transducer coupling profiles/bias^279^ The effects of the chemokine CXCL17 have been contradicted by 2 other studies^274^^,^^280^
*GPR37*	Prosaptide^281^ neuroprotectin D^282^	Neuropeptide head activator^283^ Prosaptide TX14^282^^,^^284^ chlorogenic acid^285^	-	Prosaptide and TX14 have both been reported as agonists of *GPR37L1* Although it was reported that mammalian tissues contain a peptide of identical amino acid sequence to Hydra head activator,^286^ there is currently no evidence for the presence of a gene encoding this peptide in any sequenced genome. However, the C-terminal tripeptide sequences of head activator and prosaptide are similar, which may explain the activity of head activator on *GPR37* Neuroprotectin D has been reported as an agonist at BLT_1_ receptors^287^ Binding of TX14 and neuroprotectin D, but not RvE1, was reported to recombinant *GPR37*^282^
*GPR37L1*	Prosaptide^281^^,^^284^^,^^288^ maresin 1^289^	-	-	Maresin 1 is also reported to activate LGR6^290^
*GPR50*	Little-LEN bound to recombinant GPR50 in a cross-linking assay and inhibited cAMP formation^291^	-	-	Mice with disrupted *GPR50* showed reduced body weight and lower gains on high-fat diet, increased levels of exercise,^292^ as well as an impaired social recognition^293^ Reported to selectivity inhibit MT_1_ receptor, not MT_2_ receptor, function upon coexpression^79^ Mitophagy may involve recruitment to the mitochondria^293^ A ligand-free cryo-EM structure of GPR50 has been described^294^
*GPR61*	A complex mix of plasmalogens evoked phosphorylation of ERK and protein kinase B in mouse cultured hippocampal neurones that could be inhibited with shRNA directed against *GPR61*^140^ 1-(1Z-octadecenyl)-2-oleoyl-*sn*-glycero-3-phosphoethanolamine^295^	-	Compound 1 was reported to act as an inverse agonist^296^ Compound 15 was visualized bound to an intracellular site and acted as an inverse agonist^297^	-
*GPR63*	S1P^298^^,^^299^ dioleoylphosphatidic acid^298^	-	Fingolimod appeared to function as an antagonist^299^	S1P activation was not replicated.^20^ Unlike the *Xenopus* orthologue, mammalian *GPR63* was not responsive to LysoPA^300^
*GPR75*	CCL5/RANTES^301^^,^^302^ 20-HETE^303^^,^^304^	(2-[[(5*Z*,14*Z*)-20-hydroxyicosa-5,14-dienoyl]amino]acetic acid^305^	CCL5/RANTES^304^ 19-HEDE^306^ Limonoid walrobsin A^307^ N-disodium succinate-20-hydroxyeicosa-6 (Z), 15 (*Z*)- diencarboxamide (AAA)^308^	Surface plasmon resonance provided evidence for 20-HETE binding to *GPR75*^304^ 20-HETE or CCL5 failed to evoke signaling with recombinant *GPR75* in one report^309^ A ligand-free cryo-EM structure of GPR75 has been described^310^
*GPR83*	Zn^2+^^311^ PEN^39^^,^^312^, ^313^, ^314^ FAM237A^315^, ^316^, ^317^ FAM237B^317^	CPD1, CPD27^318^	CPD25^318^	One study observed responses to FAM237A in the absence of responses to PEN with recombinant *GPR83*^316^ A further report failed to provide evidence for PEN-evoked responses in *GPR83*-expressing cells or to exhibit specific binding of iodinated PEN
*GPR87*	LysoPA^319^, ^320^, ^321^	-	Ki16425 and dioctanoylglycerol pyrophosphate^319^	In comparison with LysoPA-evoked signaling via LPA_1_ receptors, LysoPA-evoked signaling through *GPR87* was much reduced, and specific binding of ^3^H-LPA was not apparent^322^
*GPR101*	GnRH-1-5^323^^,^^324^ N-3 Docosapentaenoic acid–derived resolvin D_5_^325^	AA-14^326^	-	-
*GPR139*	L-Phe and L-Trp^327^, ^328^, ^329^ ACTH, *α*-MSH, and *β*-MSH^39^^,^^328^	LP-360924^330^ compound 1a^331^ (S)-3-chloro-N-(2-oxo-2-((1-phenylethyl)amino)ethyl)benzamide Compound 7c^332^ JNJ63533054^327^ TC0931^333^ JNJ63533054^334^ AC4^335^ zelatriazin (TAK-041)^336^ JNJ63533054^337^ Compound 6^338^	LP-471756^330^ NCRW0105-E06^338^^,^^339^ JNJ3792165^333^^,^^338^ NCRW0005-F05^338^	Activation by ACTH, *α*-MSH, and *β*-MSH was not replicated in a later study^333^ Recombinant *GPR139* has been labeled with ^3^H-JNJ63533054^327^^,^^333^ A cryo-EM structure of an agonist-bound *GPR139* has been described^337^
*GPR142*	L-Phe^340^ L-Trp^341^	Compound 33^342^ Compound 22^343^ Compound 22^344^ Compound 33^345^ Compound 40^346^ Compound 20e^347^ LY3325656^348^		
*GPR146*	Proinsulin C-peptide^349^ Cholesin C7orf50^350^			A study using *GPR146* revealed no response to C-peptide at concentrations of up to 33 *μ*M^351^
*GPR151*	Galanin^352^ Reported to be activated at acidic pH^353^	-	-	A later study was unable to observe responses to galanin^39^
*GPR160*	CARTp^354^	-	-	The suggestion that CARTp activates or binds to *GPR160* has not been supported in two subsequent studies^355^^,^^356^
*GPR162*	CART(55–102)^357^	-	-	-
*GPR171*	BigLEN^358^	MS0015203^359^	MS0021570_1^360^	-
*GPR173*	GnRH-1-5^361^^,^^362^ Phoenixin-14^363^, ^364^, ^365^ Phoenixin-20^364^^,^^366^ CCK-4 and CCK-8^367^	-	Compound 1a is reported as an inverse agonist^150^ devazepide and loxiglumide^367^	
*GPR174*	LysoPS^368^, ^369^, ^370^ CCL21^371^	Compound 12^370^	Compound 7d^372^	Cryo-EM structures of LysoPS bound to *GPR174* have been reported^259^^,^^373^^,^^374^
*MAS1L*	Angiotensin II, angiotensin IV, and Ang-(1–7)^210^	-	-	-
*MRGPRE*	A conjugate of tryptophan linked to cholic acid generated by the gut microbiome^375^	-	-	-
*MRGPRX2*	Cortistatin^4^^,^^376^^,^^377^ PAMP-20^377^, ^378^, ^379^, ^380^ LL-37^381^ *β*-Defensins^382^ ATRA^383^ CXCL14^379^ CXCL17^384^ Competence-stimulating peptide 1^380^	PMX-53^385^ complanadine A^386^ ZINC-3573^387^ compound 48/80^380^^,^^388^^,^^389^ Murepavadin^390^	Compound 1 and compound 2^391^ GE1111^392^^,^^393^ PSB172656^379^^,^^394^	*MRGPRX2* appears to respond to a wide range of basic compounds
*MRGPRX4*	Deoxycholic acid and chenodeoxycholic acid^395^^,^^396^ Lithocholic acid^397^ Bilirubin^398^	PSB22034, PSB22040^399^	HEP50768^400^	A cryo-EM structure of 3-sulfodeoxycholic acid bound to *MRGPRX4* has been reported^401^ Deoxycholic acid was ineffective in a *β*-arrestin screen of *MRGPRX4*^4^ *MRGPRX4* orthologs are not found in rodents
*P2RY8*	S-Geranylgeranyl-l-glutathione^402^	-	-	*P2RY8* orthologs are not found in rodents
*P2RY10*	LysoPA and S1P^403^ LysoPS^39^^,^^368^, ^369^, ^370^	9-o-C11^370^ Compounds 22, 33, and 34^404^ Compound 3r^405^	Dioctanoylglycerol pyrophosphate^403^ JTE-013^403^	A later study was unable to observe responses to LysoPA^39^

5HIAA, 5-hydroxyindoleacetic acid; 19-HEDE, 19-hydroxyeicosa-5(Z) 14(Z)-dienoic acid; 20-HETE, 20-hydroxyeicosatetraenoic acid; ACTH, adrenocorticotropic hormone; ATRA, All-*trans*-retinoic acid; OEA, *N*-oleoylethanolamine; ODA, oleamide; LysoPA, lysophosphatidic acid; LysoPS, lysophosphatidylserine; ML145, CID2286812, 2-hydroxy-4-[4-[(5Z)-5-[(E)-2-methyl-3-phenylprop-2-enylidene]-4-oxo-2-sulfanylidene-1,3-thiazolidin-3-yl]butanoylamino]benzoic acid; MSH, melanocyte-stimulating hormone; RvD1, resolvin D1; SPC, sphingosylphosphorylcholine.

Analysis of the putative ligands for this group of orphan GPCRs suggests 3 major chemical classes: lipids and their derivatives, peptides, and other metabolites.

Multiple orphan GPCRs have been suggested to respond to lipids, including the lysophospholipids sphingosine 1-phosphate (S1P) (*GPR3*, *GPR6*, *GPR12*, *GPR63*, and P2RY10) and lysophosphatidic acid (*GPR35*, *GPR87*, and P2RY10) (Table 6). These 2 endogenous lysophospholipids activate 2 families of receptors that have some overlapping sequence similarities, such that S1P_1_, S1P_2_, S1P_3_, S1P_4_, S1P_5_, and LPA_1_, LPA_2_, and LPA_3_ receptors were initially described as a protein family derived from 8 endothelial differentiation genes.^7^, ^8^, ^9^ However, later-identified members of the LPA receptor family, LPA_4_ and LPA_6_, were sufficiently different in sequence that they were initially proposed as members of the P2Y receptor family.^10^^,^^11^ This illustrates the observation that although sequence similarities are useful as preliminary guides for nomenclature, the overriding factor in determining nomenclature must be pharmacological evidence.^12^

Other lipid derivatives suggested to activate orphan GPCRs in this group include fatty acids (*GPR3*, *GPR6*, and *GPR12*), lysophosphatidylserine (*GPR34*, *GPR174*, and P2RY10), phospholipids and their derivatives (*GPR61* and *GPR63*), and a variety of oxidized fatty acid metabolites, including 20-hydroxyeicosatetraenoic acid (*GPR75*), lipoxin A4 (*GPR32*), maresin 1 (*GPR37L1*), neuroprotectin D1 (*GPR37*), and resolvin analogs (*GPR32* and *GPR101*). Ring-based lipids, including isoprenoid derivatives (*MRGPRX2* and all-*trans*-retinoic acid; *P2RY8* and *S*-geranylgeranyl-l-glutathione) and steroid derivatives (*MRGPRE* and a cholic acid conjugate; *MRGPRX4* and cholic acid derivatives), are also reported to activate orphan GPCRs in this group.

Two GPCRs (*GPR139* and *GPR142*) have been proposed to respond to aromatic amino acids, whereas several other orphan GPCRs have been suggested to be activated by oligopeptides active at established GPCRs, ranging from CCK-4 (*GPR173*) and the pentapeptide metabolite of gonadotropin-releasing hormone (*GPR101* and *GPR173*), to various metabolites of angiotensin II (*MAS1L*) and galanin (*GPR151*). Three orphan GPCRs (*GPR25*, *GPR35*, and *MRGPRX2*) have been reported to respond to the endogenous chemokine-like mediator CXCL17, whereas other chemokines have been reported to activate *GPR75* and *GPR174* (Table 6).

Other, less well-characterized peptides have been reported to activate *GPR19*, *GPR37*, *GPR37L1*, *GPR83*, *GPR146*, *GPR160*, *GPR162*, *GPR171*, *GPR173*, and *MRGPRX2* (see Table 6).

The remaining candidate endogenous ligands could be loosely grouped as metabolites, ranging from the inorganic agents, protons (*GPR151*) and zinc ions (*GPR83*) to amino acid and monoamine metabolites (*GPR35*) and bilirubin (*MRGPRX4*).

Based on these data, it would be a recommendation from NC-IUPHAR for further independent investigation of these preliminary pairings; of particular focus would be an assessment of the colocalization of patho/physiologically relevant concentrations of the putative endogenous agonist with the target GPCR.

## V Orphan class A G protein-coupled receptors with substantive evidence supporting endogenous/physiological ligand identification

For this group of class A GPCRs, the expert reviewers completed initial, independent assessments, which led to discussions across the whole group to reach a consensus, which evaluated them as stronger candidates for deorphanization (Table 7). As with the previous group lacking convincing evidence for deorphanization (Table 6), the putative endogenous ligands could initially be subdivided based on chemical classes.

**Table 7:** Ten human class A orphan G protein-coupled receptor evaluated for orphan status and identified as having high potential for deorphanization at the current time

Target	Proposed Endogenous/Physiological Agonist/s	Surrogate Agonist/s	Antagonists/Inverse Agonists	Comment
*GPR4*	Protons^48^^,^^49^^,^^406^, ^407^, ^408^	-	Compound 1^409^ GPR4 antagonist 3b^410^ Compound 39c^411^ NE 52-QQ57^412^, ^413^, ^414^	Cryo-EM structures have been reported^54^^,^^415^, ^416^, ^417^, ^418^
*GPR15*	GPR15L^37^^,^^38^^,^^40^^,^^419^ GPR15L (71-81)^39^^,^^419^	-	Antibodies directed against *GPR15* were described to block GPR15L-mediated T-cell recruitment^420^	Binding of modified GPR15L to *GPR15* has been reported^38^^,^^40^^,^^419^^,^^421^ A cryo-EM structure of GPR15 with GPR15L bound has been described^422^ GPR15L has also been reported to activate CXCR4 in vitro^423^
*GPR31*	12S-HETE^13^^,^^14^^,^^424^^,^^425^	SB583831 and SB583355^21^	SAH2^424^	Radiolabeled 12S-HETE binding to recombinant *GPR31* has been described^13^^,^^425^ 12S-HETE was ineffective in a *β*-arrestin screen of *GPR31*^4^
*GPR39*	Zn^2+66,67,70,^^426^	TC-G 1008^427^ compound 15^428^ Cpmd1324^429^^,^^430^	VC43^431^ Compound 61^432^	Knockdown of *GPR39* in human HaCaT keratinocytes attenuated zinc-evoked responses^69^ A PET ligand for GPR39 in vivo has been described^433^
*GPR65*	Protons^48^^,^^50^^,^^408^^,^^434^	-	Psychosine appeared to act as an agonist at the recombinant receptor,^435^ although a later report suggested antagonist action at acid-induced signaling^50^	BTB09089 was initially reported as a positive allosteric modulator of *GPR65,*^436^^,^^437^ and then later suggested to target RhoA instead^438^
*GPR68*	Protons^48^^,^^49^^,^^408^^,^^436^^,^^439^^,^^440^	-	Ogremorphin^441^ Compound 18l^442^	MS48107,^443^ ogerin,^436^ and lorazepam^55^^,^^436^ have been described as positive allosteric modulators A cryo-EM structure has been described^418^
*GPR132*	9-HODE^19^^,^^21^^,^^25^^,^^444^	ONC212^445^ CP55940, SKF95667, SB583831, and SB583355^21^ NOX-6-7^25^ T10418^444^	GSK1820795A^21^ NOX-6-18^25^	9-HODE, *N*-palmitoylglycine, and *N*-linoleoylglycine were ineffective in a *β*-arrestin screen of *GPR132*^4^ Crystal structures of *GPR132* have been described with bound *N*-palmitoylglycine or 9 (S)-hydroxy-10,12-octadecadienoic acid^25^
*GPR183*	7*α*,25-dihydroxycholesterol^31^, ^32^, ^33^^,^^446^, ^447^, ^448^, ^449^ 7*α*,27-OHC, 7*α*-OHC, 7*β*-OHC, 7*β*, 27-OHC, 7*β*, 25-OHC, 25-OHC, OHC^31^^,^^32^^,^^446^	NIBR51^448^ T10418^444^	GSK682753A^450^^,^^451^ NIBR189^448^ SAE14^452^ compound 32^453^ compound 33^447^	Radiolabeled 7*α*,25-diOHC binding to recombinant *GPR183* has been described^31^^,^^32^^,^^450^ Cryo-EM structures of *GPR183* bound with GSK682753A and 7*α*,25-diOHC have been reported^454^
*MRGPRD*	*β*-alanine^4^^,^^56^, ^57^, ^58^^,^^455^ Alamandine^60^	Diethylstilbestrol^58^ EP2825 and EP3945^455^	MU6840^58^ PD123319^60^ D-Pro^7^-Ang-(1–7)^62^	Alamandine and *β*-alanine displaced binding of a fluorescently labeled alamandine peptide in recombinant *MRGPRD* expression^59^ Cryo-EM structures have been reported of *MRGPRD* bound with EP2825 and EP3945^455^
*MRGPRX1*	Bovine adrenal medulla peptide 8-22^4^^,^^42^, ^43^, ^44^, ^45^	Chloroquine^456^ compound 16^457^^,^^458^ FSLLRY^459^ fungal micasin^460^	Berbamine^459^^,^^461^	ML382 is reported as a positive allosteric modulator of the receptor.^43^ Cryo-EM structures of *MRGPRX1* bound with BAM8-22 have been described,^458^^,^^462^ as well as the synthetic agonist compound 16^463^

OHC, hydroxycholesterol.

### A Potential lipid-sensing G protein-coupled receptors: GPR31, GPR132, and GPR183

For all 3 of these GPCRs, synthetic agonists and antagonists have been described, which should facilitate the broader characterization of pathophysiological signaling.

***GPR31***: 12S-Hydroxyeicosatetraenoic acid (12S-HETE, (5*Z*,8*Z*,10*E*,12S,14*Z*)-12-hydroxyeicosa-5,8,10,14-tetraenoic acid) is derived from arachidonic acid through 12S-lipoxygenase activity. 12S-HETE was initially described to activate *GPR31* in recombinant systems in 2011.^13^ The authors investigated the potential for binding of a radiolabeled version, which they observed to have high affinity (pK_d_ value of 8.3). Functional analysis of these cells identified 12S-HETE-enhanced binding of guanosine 5′-*O*-(3-[^35^S]thio)triphosphate with a potency of 9.5 (pEC_50_ value) and activation of extracellular signal-regulated kinase. In an independent analysis, 300 nM 12S-HETE inhibited cAMP-mediated reporter gene expression in CHO cells expressing human recombinant *GPR31*.^14^

In a model of liver ischemia-reperfusion injury, symptoms were abated in an identical manner by interfering with either 12S-lipoxygenase or *GPR31*.^15^

It should be noted that lactate and pyruvate have also been suggested to activate *GPR31*,^16^, ^17^, ^18^ and knockdown of *GPR31* in human intestinal dendritic cells attenuated pyruvate-evoked responses.^16^

Although the evidence for activation by 12S-HETE appears consistent in mouse and human recombinant receptors, evidence for the protein expression of *GPR31* in human tissues is limited.

***GPR132***: Particular oxylipins, oxidized metabolites of fatty acids, increased guanosine 5′-*O*-(3-[^35^S]thio)triphosphate or binding, inositol phosphates accumulation, and elevated intracellular calcium in cells expressing recombinant *GPR132*.^19^ Specifically, 9(S)-hydroxyoctadecadienoic acid (9S-HODE), derived from linoleic acid, and 11-hydroxy-5,8,12,14-eicosatetraenoic acid, derived from arachidonic acid, are possible agonists. The potency of 9S-HODE for calcium mobilization was ∼5.6 (pEC_50_ value). 9S-HODE and 11-hydroxy-5,8,12,14-eicosatetraenoic acid were confirmed as agonists in independent studies using recombinant *GPR132,* measuring *β*-arrestin accumulation,^20^ and a yeast pheromone gene assay replicated the potency of 9S-HODE with a pEC_50_ value of 5.9.^21^

One reservation noted was that the reported plasma concentration of 9-hydroxyoctadecadienoic acid was 58 nM in healthy rats,^22^ although inflammation increased tissue levels in the sciatic nerve from ∼150 to over 400 pmol/mg tissue.^23^

*GPR132* has also been reported to be activated by amino acid conjugates of fatty acids, including *N*-(3-hydroxypalmitoyl)glycine,^24^ *N*-palmitoylglycine,^21^^,^^25^ and *N*-linoleoylglycine,^21^ although cryo-EM studies suggested a different binding profile compared with oxidized fatty acids.^25^

There is contradictory literature about the impact of lysophospholipids on *GPR132* function. Responses to lysophosphatidylcholine (LysoPC) in isolated human neutrophils were blocked by an antibody directed at *GPR132*,^26^ whereas a report that LysoPC and sphingosylphosphorylcholine activated *GPR132* has been retracted,^27^ as has a report suggesting activation of *GPR132* by LysoPC.^28^ Multiple reports suggest that LysoPC^26^^,^^28^ and LysoPS^26^ can activate *GPR132* in vitro. LysoPC was also reported to block the effects of protons on *GPR132* signaling.^29^ Whether these actions of lipid metabolites are mediated through an orthosteric or allosteric mechanism (see discussion below for *GPR68*) awaits further investigation.

***GPR183***: Oxysterols are a family of sterol derivatives, among which 7*α*,25-dihydroxycholesterol (7*α*,25-diOHC) is formed by the sequential hydroxylation of cholesterol by cholesterol 25-hydroxylase and CYP7B1.^30^ Subsequent turnover of 7*α*,25-diOHC is mediated by hydroxyl-*Δ*-5-steroid dehydrogenase, 3*β*- and steroid *Δ*-isomerase 7, leading to the production of bile acids.^30^ Two publications in 2011 identified that 7*α*,25-diOHC activated recombinant *GPR183*^31^^,^^32^ with potencies (pEC_50_ values) of 8.9 (guanosine 5′-*O*-(3-[^35^S]thio)triphosphate binding) and 8.7 (calcium mobilization).

^3^H-7*α*,25-diOHC was observed to bind specifically to the recombinant receptor by different groups with a range of *K*_d_ values of 450 pM^32^ to 24 nM.^33^

Modification of the expression of cholesterol 25-hydroxylase, a synthetic enzyme for oxysterols, altered signaling through *GPR183*.^31^^,^^34^, ^35^, ^36^

### B Potential peptide-sensing G protein-coupled receptors: GPR15 and MRGPRX1

For these GPCRs, there are limited examples of synthetic agonists and antagonists, which will hamper further characterization of these receptors.

***GPR15***: A highly cationic 81-aa polypeptide with chemokine-like properties, isolated from pig gut, activated recombinant GPR15 in calcium mobilization assays.^37^ A truncated peptide identified as GPR15L-(25-81) exhibited a potency (pEC_50_ value) of ∼7.5 in this assay. An independent investigation, building on a patent disclosure, described the potency of full-length “GPR15L” in a *GPR15*-*β*-arrestin assay of 7.7.^38^ Other fragments of or modified peptides from “GPR15L” were reported to activate recombinant *GPR15* in assays of cAMP inhibition with a potency of 6.7^39^ or ERK activation with a potency of ∼8.2.^40^

A fusion protein of “GPR15L” with the fragment crystallizable portion of human IgG bound specifically to cells expressing recombinant *GPR15*, but not to untransfected cells. The potency (pIC_50_ value) for “GPR15L” displacement of fusion protein binding was ∼7.0.^38^

Fragments of cystatin C have been proposed to activate GPR15, although through a distinct mechanism of action.^41^

Previous guidance on receptor nomenclature from NC-IUPHAR recommended that a receptor be named after its endogenous agonist.^1^^,^^12^ The potential re-naming of GPR15 is complicated, however, by the sequence of discovery, where an endogenous peptide was identified after the receptor was identified and named after the GPCR.

***MRGPRX1***: Proenkephalin A is a protein precursor of the endogenous opioid pentapeptide enkephalins but also produces a 22-aa fragment called bovine adrenal medulla peptide 22, which was found to activate the *δ*-opioid peptide receptor in vitro, as well as *MRGPRX1*.^42^ A truncated version, bovine adrenal medulla peptide 8-22 (BAM-8-22), exhibited selective activation of *MRGPRX1* for calcium mobilization, with a potency (pEC_50_ value) of 7.5, without activity at *δ* opioid peptide receptor up to micromolar concentrations.^42^ Activation of *MRGPRX1* by BAM-8-22 has been reproduced in subsequent independent investigations.^4^^,^^43^, ^44^, ^45^

Specific binding of ^3^H-BAM-8-22 was observed using recombinant *MRGPRX1* with a pK_d_ value of ∼8.0.^42^ Chemokine CXCL17 has been reported to activate *MRGPRX1* in vitro.^46^

Peptide fragments of hemoglobin (hemorphins LVV-H7 and VV-H7) were identified in human platelets and activated recombinant MRGPRX1, albeit with much lower potency than BAM-8-22.^47^

Studies in rodents are confounded because they lack orthologues of this receptor, so it will be challenging to investigate the manipulation of proenkephalin A expression and its influence on *MRGPRX1* activity.

### C A potential family of proton-sensing G protein-coupled receptors: GPR4, GPR65, and GPR68

In normal plasma and extracellular fluids, pH is tightly regulated, but in inflammatory states and with some cancers, extracellular pH can drop below 7.3, which may have implications for signal transduction. At the time of writing, these 3 GPCRs have a limited repertoire of synthetic pharmacological tools, so an aspiration of this review is that more widespread attention on these receptors should facilitate drug discovery in this area.

*GPR4*, *GPR65*, and *GPR68* constitute a subfamily with higher homology to each other in comparison to the remaining class A GPCRs. One common feature is a series of 3–5 extracellular histidine residues combined with a triad of “buried” acidic glutamate and aspartate residues.^48^ It is suggested that superficial histidines act as proton sensors, leading to intramolecular rearrangements allowing the deeper acidic residues to promote coupling to G proteins, although the 3 orphans appear to have slightly different thresholds for proton activation. As indicated in Table 7, multiple independent reports support the influence of pathophysiological changes in extracellular pH as the stimulus for coupling of these 3 receptors to elevation of intracellular cAMP in the case of *GPR4*^49^ and *GPR65*^50^ or to calcium and cAMP signaling by *GPR68*.^49^^,^^51^

Membrane lysophospholipids have been suggested to influence the function of this family of GPCR, although there is a lack of clarity in terms of whether these effects are consistent and/or a mechanism of allosteric regulation. For example, a report of *GPR4* activation by sphingosylphosphorylcholine and LysoPC has been retracted^27^ as has a report that sphingosylphosphorylcholine activated *GPR68*.^52^ Lysophosphatidic acid activation of the LPA_1_ receptor has been reported to facilitate *GPR68* action in vitro.^53^ A recent cryo-EM study suggested that LysoPC bound to GPR4 allosterically to enhance receptor function.^54^

*GPR68* was also reported to respond to a number of endogenous peptides: OSTN (115-133), CARTPT (76-96), and Pomc (141-162).^39^ This report has not yet been reproduced. Sulazepam was described to act as a low-potency inverse agonist for GPR68.^55^

### D MRGPRD

*β*-Alanine is a naturally occurring alternative amino acid in mammalian systems, resulting from pyrimidine degradation, which has also been of interest as a potential anabolic dietary supplement and precursor for the dipeptide carnosine. *β*-Alanine evoked calcium mobilization and inhibition of cAMP formation in *MRGPRD*-expressing cells with a potency (pEC_50_ value) of 4.8.^56^ Responses of *MRGPRD* to similar concentrations of *β*-alanine were confirmed by several subsequent independent investigations.^4^^,^^57^^,^^58^

Decarboxylation of the amino terminus of angiotensin-1-7 converts aspartate to alanine, producing a heptapeptide named alamandine. It is suggested to be formed in the heart from Ang-1-7 metabolism and to be present in the blood.^59^ In cells expressing *MRGPRD*, alamandine evoked cAMP formation with a bell-shaped profile, in which the stimulatory phase had a potency (pEC_50_ value) of 12.4.^60^ Although alamandine has biological effects in preparations in which *MRGPRD* is expressed and is sensitive to a reported *MRGPRD* antagonist^61^, ^62^, ^63^, ^64^, the pairing does not appear to have been reproduced with the recombinant receptor.

There remains, therefore, a need for triangulation of observations, as well as the demonstration of pathophysiological levels of the putative endogenous agonists for *MRGPRD*.

### E GPR39

Zinc is an essential trace element, with multiple transporters identified for translocation of zinc ions in mammalian cells, and an estimated 10% of the human proteome has been identified as zinc-binding proteins.^65^ Extracellular concentrations of zinc ions in the micromolar range were found to enhance inositol phosphates and cAMP generation in *GPR39*-expressing cells with potencies (pEC_50_ values) of 4.7 and 5.1, respectively.^66^ A contemporaneous report identified that fractions of FBS that activated *GPR39*-expressing cells could be eliminated in the presence of EDTA.^67^ In these cells, micromolar zinc ion concentrations evoked rapid intracellular calcium ion elevations. *GPR39* activation by zinc ions has been proposed as a result of synaptic activity^68^ or damage.^69^

The suggested locus for zinc ion binding to *GPR39* is through 2 N-terminal histidine residues.^70^ Although this location for a canonical orthosteric ligand would be unique for a class A GPCR, the possibility has been raised that it is consistent with an allosteric ligand.

It has been reported that a fragment from the ghrelin precursor called obestatin was an agonist for *GPR39*.^71^ However, several independent groups have been unable to replicate this finding.^66^^,^^72^, ^73^, ^74^ The original authors have since confirmed that they could not reproduce their findings on obestatin binding and activation of *GPR39* in vitro.^75^

Two alternative, potentially orthosteric oxylipin agonists have been proposed for *GPR39*: 15-HETE (15-hydroxyeicosatetraenoic acid) and 14,15-epoxyeicosatrienoic acid.^76^ In a search for binding proteins for 14,15-epoxyeicosatrienoic acid, an irreversible probe labeled *GPR39* in mouse vascular smooth muscle cells. Among the other oxylipins investigated, only 15-HETE was also able to evoke extracellular signal-regulated kinase activation in transfected cells.^76^ The responses to these eicosanoids were enhanced in the presence of 1 *μ*M zinc ions, a concentration that was ineffective alone. Zinc ions and 15-HETE were also synergistic when intracellular calcium ions were measured in mouse vascular smooth muscle cells. Notably, 15-HETE and 14,15-epoxyeicosatrienoic acid were ineffective in a *β*-arrestin screen of *GPR39*.^4^

To conclude, therefore, the relationship between *GPR39*, zinc ions, and eicosanoid/oxylipin analogs needs clarifying before a change in nomenclature is warranted.

## VI Conclusions

In this review, we have provided an extended evaluation checklist for NC-IUPHAR subcommittees to conduct an unbiased evaluation of the orphan status of class A GPCRs, and indeed for all receptors.

Information about 10 of the orphan class A GPCRs (Table 7) is sufficiently advanced that we can refer these to further subcommittees of NC-IUPHAR. We would advocate for the wider community to continue investigating these suggested pairings—independent confirmation is a fundamental in science. Cell-based assay kits are available commercially to assist in those investigations, which should facilitate the investigations of receptor pharmacology. However, there remain challenges associated with determining the levels of endogenous ligands in the close environment of the receptor. In addition, for some of the putative endogenous/physiological ligands described, the enzymatic pathways for production and transformation remain obscure.

In considering the substantial numbers of class A GPCRs lacking a putative endogenous agonist or any substantive pharmacology (Table 3), we are prompted to pose a number of questions:

*Does every GPCR need to have an endogenous agonist or physiological ligand?* As with the ACKR family of atypical chemokine receptors, the possibility exists for a singular modality (e.g., through *β*-arrestin)^3^ rather than multiple downstream signaling pathways, and so we may consider that, for some of these receptors, the “correct” combination of agonist and signaling pathway may not have been tested. A further possibility is that some receptors may only be functional as part of an oligomeric complex. For family C GPCRs, there are examples of obligate heterodimers such as the GABA_B_ receptor^77^ and the sweet taste receptor, TAS1R2/TAS1R3.^78^ Although the same profile has not been described for class A GPCRs, a heterodimer between *GPR50* and the MT_1_ receptor has been described, in which *GPR50* exerted an inhibitory effect on MT_1_ receptor function.^79^

A number of class A orphan GPCRs have been described as having high constitutive activity in recombinant expression,^80^ whether this results from the particular cellular environment or from intrinsic structural properties of these receptors will require further investigation. *GPR161* provides an example where there is evidence for autoactivation of the receptor by an intrinsic peptide sequence^81^ in a manner distinct from the proteinase-activated receptors.^82^

*Does every GPCR need to respond to a single endogenous ligand (or ligand family)?* For the cannabinoid receptors, there are 2 distinct families of endogenous ligands, based on esters, such as 2-arachidonoylglycerol, and amides, such as anandamide,^83^ whereas opioid receptors have multiple endogenous peptide agonists.^84^ The nomenclature for such examples will need to be resolved on a case-by-case basis.

Additionally, there are examples of GPCRs for which the physiological agonist is derived from exogenous sources. For example, fMet-Leu-Phe is a bacterial product that activates formylpeptide receptors and prompts chemotaxis in neutrophils.^85^ Similar GPCRs in the mouth and nasal passages, the taste and odorant receptors, would be expected to respond to dietary and environmental stimuli, although they are also expressed in internal organs separated from the exterior.^86^

*Does every species orthologue need to respond to the same endogenous ligand?* Certainly, there are “complications” of evolution, which imply that orthologues may not be highly conserved across species, leading to distinct pharmacology. This is most obviously evident in the bitter taste receptors^86^ and the Mas-related GPCR family,^87^ where different receptor repertoires and distinct pharmacology are evident in human and rodent species.

In summary, although advances are being made in our understanding of the pharmacology, physiology, and biochemistry of these incredibly useful drug targets, it is apparent that there are still major opportunities for the future exploitation of these class A GPCRs.

## Conflict of interest

Authors Stephen P.H. Alexander, Kirstie A. Bennett, Elena Faccenda, Julien Hanson, Paul A. Insel, Simon D. Harding, Eamonn Kelly, Kirill A. Martemyanov, Rick R. Neubig, Stefan Offermanns, Gunnar Schulte, Nicola J. Smith declare no conflict of interest. Andrew J. Brown was an employee of GlaxoSmithKline at the time of writing the manuscript and provides consultancy services to Rewind Therapeutics, Schrodinger, Linchpin.Bio, and Mironid. Michelle Glass is a consultant for Aelis Farma Ltd. David E. Gloriam is a part-time employee and warrant holder of Kvantify. Christopher J. Langmead holds shares in GlaxoSmithKline Ltd, Pacalis Therapeutics Pty Ltd and Phrenix Therapeutics Inc. He is also a part-time employee of Phrenix Therapeutics Pty Ltd and is the recipient of research funding from Servier. Pacalis, Phrenix, and Servier are all engaged in GPCR drug discovery. Christopher J. Langmead has also received payment for consultancy from Neumora Therapeutics Inc and Curie.Bio. Mette M. Rosenkilde is co-founder and minor shareholder of Antag Therapeutics and Synklino, and consultant at Aikium. Huixian Wu is an employee of Pfizer Inc. Anthony P. Davenport is a member of scientific advisory boards on endothelin for Johnson & Johnson, ENB Therapeutics and Pharmazz.
